# The challenge of benchmarking health systems: is ICT innovation capacity more systemic than organizational dependent?

**DOI:** 10.1186/s13584-015-0036-5

**Published:** 2015-08-19

**Authors:** Luís Velez Lapão

**Affiliations:** Global Health and Tropical Medicine, WHO Collaborating Center for Health Workforce Policy and Planning, Instituto de Higiene e Medicina Tropical, Universidade Nova de Lisboa, Lisboa, Portugal

**Keywords:** ICT, eHealth, Health policies, Complexity, Benchmarking, Innovation

## Abstract

The article by Catan et al. presents a benchmarking exercise comparing Israel and Portugal on the implementation of Information and Communication Technologies in the healthcare sector. Special attention was given to e-Health and m-Health. The authors collected information via a set of interviews with key stakeholders. They compared two different cultures and societies, which have reached slightly different implementation outcomes. Although the comparison is very enlightening, it is also challenging.

Benchmarking exercises present a set of challenges, such as the choice of methodologies and the assessment of the impact on organizational strategy. Precise benchmarking methodology is a valid tool for eliciting information about alternatives for improving health systems. However, many beneficial interventions, which benchmark as effective, fail to translate into meaningful healthcare outcomes across contexts. There is a relationship between results and the innovational and competitive environments.

Differences in healthcare governance and financing models are well known; but little is known about their impact on Information and Communication Technology implementation. The article by Catan et al. provides interesting clues about this issue. Public systems (such as those of Portugal, UK, Sweden, Spain, etc.) present specific advantages and disadvantages concerning Information and Communication Technology development and implementation. Meanwhile, private systems based fundamentally on insurance packages, (such as Israel, Germany, Netherlands or USA) present a different set of advantages and disadvantages - especially a more open context for innovation.

Challenging issues from both the Portuguese and Israeli cases will be addressed. Clearly, more research is needed on both benchmarking methodologies and on ICT implementation strategies.

## Background

The study by Catan et al. [1] uses an exploratory approach, fundamentally to perform a benchmarking exercise comparing Israel and Portugal on the implementation of Information and Communication Technologies (ICT) in the healthcare sector. E-Health and m-Health implementation approaches naturally received special attention.

The authors’ primary aim was to compare two different cultures and societies, which have reached slightly different ICT implementation results, so as to draw lessons for improved policy-making. Although the comparison of two different contexts can be very enlightening, it is also a challenging process.

The authors collected benchmark information by interviewing a set of stakeholders from the two countries. Methodologically, there was an imbalance between the two countries in terms of the range of stakeholders interviewed; the Portuguese side was not so well covered, and did not reach theoretical saturation. Another interesting difference is that one could also find a sense of “optimism” from the Israelis interviewed as well as a sense of “pessimism” from the Portuguese side. This difference could be related more to a cultural perspective than to a real difference in ICT implementation.

Both Israel and Portugal are amongst the most developed countries concerning ICT use in the public sector [2]. In the health sector, in particular, Portugal, like the UK, with a National Health Service founded in 1979, has been able to develop national ICT systems that can be found in most hospitals and health centers [3, 4]. Portugal has a national electronic healthcare system (not yet fully operational) and other innovative solutions such as the national system to manage surgical queues [5].

## Benchmarking complex health systems

Health systems are complex, making comparisons particularly challenging; this is especially the case when the countries involved share few similarities [6]. Other well-known aspects are the socio-technical factors, or “fit” factors, that complicate the design and deployment of health information systems [7]. These factors are often not taken in consideration, leading to significant system implementation failures [8]. Catan et al. did not look at these socio-technical factors (implementation strategies, participative design, usability, autonomy, etc.). They have instead addressed patient empowerment. Patient empowerment is a stimulating, but still young, concept. New research on the topic shows how delicate it is [9]. It depends on complex issues such as the patient’s attitudes and various healthcare management and organizational factors.

Benchmarking exercises also need to cope with complexity in health services. Benchmarking is often used as a process of comparing one’s organizational processes and performance metrics to sector best practices from other organizations [2]. Comparing healthcare ICT in Israel and Portugal could represent an opportunity to provide important information for policy making, as both countries are clearly supporting a strategy towards better ICT in healthcare.

The Catan et al. article presents an exploratory study. An exploratory approach enables the identification of important issues for further study, but it does not really provide clear, value added, information for policy making [10].

## ICT as an innovation in healthcare

Innovation is another challenging subject in healthcare. Several authors consider that innovation is the most promising approach to transforming healthcare into a more productive and safer system. However, it is a common mistake to consider any ICT solution as an innovation. This is simply not the case. As innovation is the use of new ideas, or concepts, to improve processes; it requires that ICT solutions provide real value to health professionals. Unfortunately, it is well-known that wrongly deployed ICT can even seriously harm patients [11]. An example is the ICT systems that do not check for security issues like radiation dosage or medicines or even ICT that do not support well-integrated databases, mixing patient information. Hence, innovation needs to be planned and implemented very professionally.

In fact, the health sector firstly addressed the use of ICT not as an innovative process but rather as part of a search for a more effective way to control costs and financial movements. Considering ICT to provide innovative alternatives to health processes is a fairly new approach. Moreover, it is recognized that there exists little knowledge and evidence about the contribution of ICT to cost reduction [12]. As ICT policy-making is severely lacking in evidence, more studies are required to provide more evidence.

In comparing Israel and Portugal, Catan et al. failed to specify whether patient health needs were the same in both countries, and if those needs can be well (and cost-effectively) covered with e- or m-Health systems. Table one shows that, although there are relevant differences, the two countries still have many similar health indicators.

In a benchmarking exercise, analysts need also to think about whether there are other variables that could explain the differences in the responses found. For instance, do the institutions being compared have the same level of knowledge? Are the change processes being led by professionals with significantly roles in their organizations? It is completely different if there is a Chief Information Officer or a Human Resources Manager leading the process. There are also other issues, such as, what are the functions of physicians in the system, and whether there are referral mechanisms. Clearly, several context-dependent variables can promote or limit innovation in healthcare.

Benchmarking healthcare systems is a valid tool for eliciting information about alternatives for improving health systems. However, many beneficial interventions that are thought to be effective fail to translate into meaningful health care outcomes across contexts. Therefore, it is essential to properly evaluate the translation of “good practices” measures.

## Innovation in different competitive environments

Benchmarking presents a set of challenges, such as methodology choice and actual impact assessment. Rigorous benchmarking methodology can be a valid tool for eliciting about alternatives to improve health systems. However, many beneficial interventions benchmark as effective fail to translate into meaningful healthcare outcomes across contexts. The relationship between innovation and competitive environments needs to be taken in consideration. To better cope with complexity, benchmarking exercises have to be comprehensive and focused on implementation as explained bellow.

The impact of different healthcare governance and financing models are well known; but little is known about their impact on ICT implementation. The article by Catan et al. papers provides interesting clues about this matter. Public systems (such as those in Portugal, UK, Sweden, Spain, etc.) present specific advantages and disadvantages concerning ICT development and implementation. Meanwhile, private systems, based fundamentally on insurance packages (Israel, USA, Germany, Netherlands, etc.) present a different set of advantages and disadvantages - especially a more open context for innovation.

An important message is that innovation is expected to be more effective within more competitive environments. Economists have shown that large and persistent public-private differences in productivity levels can be found in many different sectors [13], including healthcare. Although public systems present advantages from bigger scale and integration, private systems seem to be more innovative [14].

Some health professionals are reluctant to adopt technological innovation, but most of them are not unalterably opposed. They just need to be properly convinced that the new technology being introduced is valuable and truly innovative. Unfortunately, technologies often fail to fulfill the needs of patients and health professionals [15]. Repeatedly, the ICT used in healthcare is of low quality, lacking usability, and not innovative at all [15]. Still, both in Portugal and Israel one can find several cases of valuable and innovative bottom-up approaches. However, though many promises ICT maturity in healthcare is yet to be reached [8].

To better cope with the complexity and the differences between the cases being benchmarked, it is vital to use rigorous approaches. Many implementation theories, such as the “Consolidated Framework For Implementation Research” (CFIR), have been developed to address implementation effectiveness [16]. The CFIR offers an overarching typology to promote implementation theory development and verify what works where and why (from benchmarking), across multiple contexts. Healthcare ICT can gain from CFIR’s realistic structure for approaching complex, interacting, multi-level, and transient states of constructs in the real world by embracing and unifying key (and comparable) constructs from implementation theory literature.

## Conclusion

Best practices are important to improve the quality of processes and decision-making in healthcare organizations. However, it requires precise methodologies, such as benchmarking. Benchmarking exercises, when done correctly, can provide significant information to promote effective best practices usage. The response to healthcare challenges could surely benefit from more exchange and better understanding of other countries’ experiences if the benchmarking process provides relevant information.

Nevertheless, best practices alone are not sufficient as, due to organizational complexity, their implementation is indeed context-dependent. Policy-makers also have to recognize, and address, the fact that innovation (e.g. an eHealth solution) is contingent on the existing competitive environment, as Catan et al. have demonstrated.

Clearly, more research is needed on both benchmarking methodologies and on ICT implementation strategies.

## Abbreviations

CFIR: Consolidated Framework For Implementation Research
ICT: Information and Communication Technologies
USA: United States of America
